# Supplementary material for: Pri-miR526b and Pri-miR655 Are Potential Blood Biomarkers for Breast Cancer
Source: Cancers (Basel). 2021 Jul 30;13(15):3838. doi: 10.3390/cancers13153838 (PMC8345356; doi:10.3390/cancers13153838)
Supplement: Supplementary file 1 [file cancers-13-03838-s001.zip › cancers-1316119-supplementary.pdf]

cancers MDPI

Supplementary

Pri-miR526b and Pri-miR655 Are Potential Blood Biomarkers for Breast Cancer

Mousumi Majumder, Kingsley Chukwunonso Ugwuagbo, Sujit Maiti, Peeyush K Lala and Muriel Brackstone

Table S1. Clinical characteristics of breast cancer subject and control subject demographics of the samples from the Ontario Institute for Cancer Research.

**Characteristics**		**Control** ***n*** **= 20**	**Breast Cancer** ***n*** **= 20 (100%)**
**Sex**	Male	0	0
	Female	20	20
**Age (years)**	Mean ± SD (range)	54.39 ± 16.95 (27-87)	67.25 ± 18.17 (32-92)
**Tumor Receptor Status**	ER+ve (%)		15 (75.00)
	ER-ve (%)		2 (11.11)
	PR+ve (%)		14 (70.00)
	PR-ve (%)		3 (15.00)
	HER2+ve (%)		3 (15.00)
	HER2-ve (%)		12 (60.00)
**T-N-M (Tumor) Staging**	0		1 (5.00)
	I		1 (5.00)
	II		13 (65.00)
	III		5 (25.00)
